# Two-channel endoscope re-intervention for stent dysfunction after interventional endoscopic ultrasonography: forceps stabilization of stent for cutting by argon plasma coagulation

**DOI:** 10.1055/a-2106-1292

**Published:** 2023-06-27

**Authors:** Haruka Toyonaga, Masayo Motoya, Tsuyoshi Hayashi, Toshifumi Kin, Kuniyuki Takahashi, Akio Katanuma

**Affiliations:** Center for Gastroenterology, Teine Keijinkai Hospital, Hokkaido, Japan

As interventional endoscopic ultrasonography (EUS) has become widely performed, occasions for re-intervention because of stent dysfunction are increasing and several re-intervention methods have been reported 1 2 3 4 5 . We introduce here a novel technique utilizing a two-channel endoscope (GIF-2TQ260M; Olympus, Japan), in which a forceps is used to hold a stent in the required position whilst it is trimmed using argon plasma coagulation (APC) ( Fig. 1 ).

**Fig. 1:**
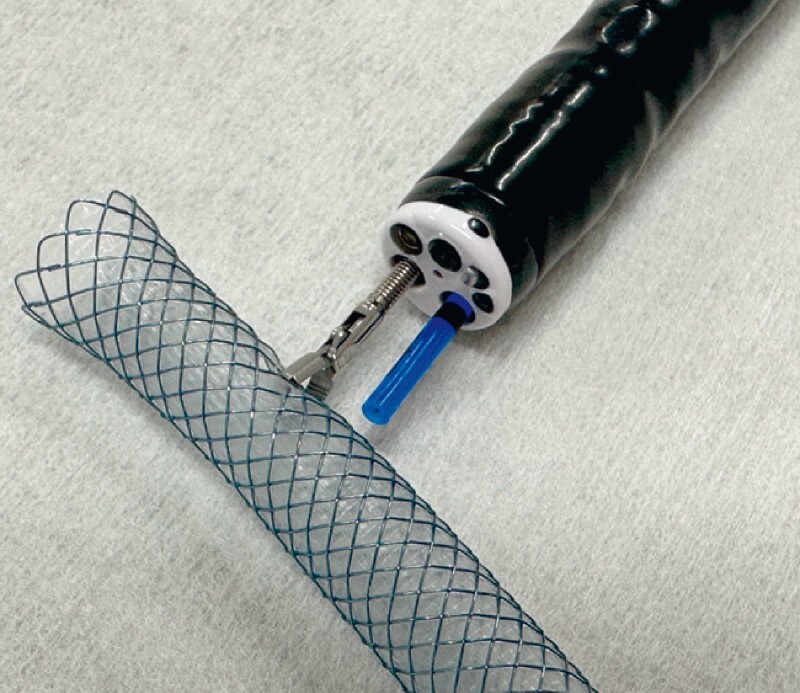
Two-channel endoscope setup. The two-channel endoscope (GIF-2TQ260 M, Olympus, Japan); a forceps can be deployed from one channel to grasp the metal stent, and an argon plasma coagulation (APC) probe can be deployed from the other channel to trim the stent.

*Case 1.* A 91-year-old woman had undergone an EUS-guided hepaticogastrostomy in which an end-bare self-expandable metal stent (SEMS) was deployed in the B3 segment. However she experienced cholangitis due to obstruction of the SEMS. For biliary access and biliary drainage, the gastric end of the stent was trimmed using APC. Use of the two-channel scope enabled grasping of the end of the SEMS with forceps to hold it in place whilst trimming with APC was done ( Fig. 2 ). By grasping the end of the stent and applying traction, efficient and rapid SEMS cutting was achieved with a stable view and fixation of the area where APC was applied ( Fig. 3 , Video 1 ).

**Fig. 2:**
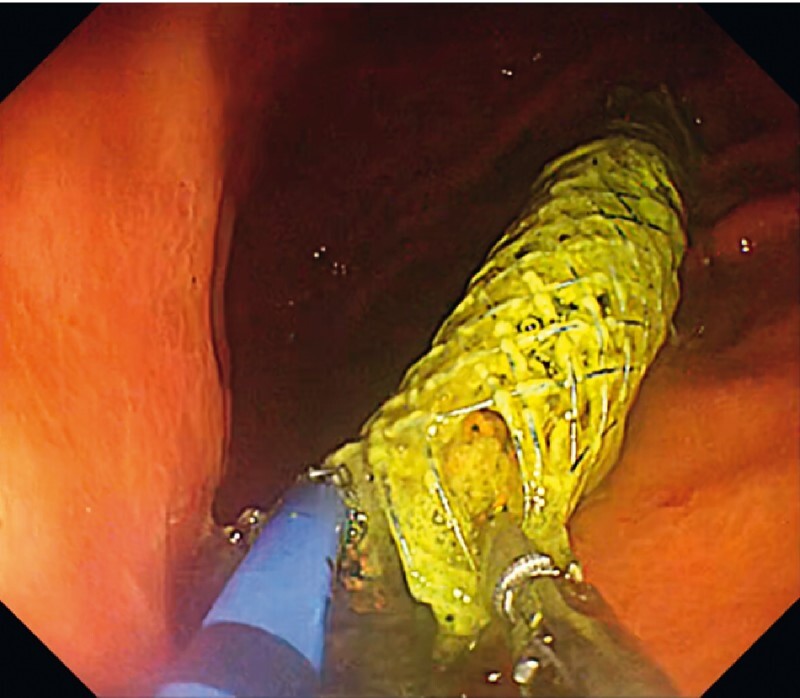
*Case 1.* Trimming of an end-bare self-expandable metal stent (SEMS) deployed in the B3 liver segment during endoscopic ultrasound (EUS)-guided hepaticogastrostomy. A two-channel endoscope is being used, so that the gastric end of the SEMS can be held in the required position with forceps whilst cutting is done by argon plasma coagulation (APC).

**Fig. 3:**
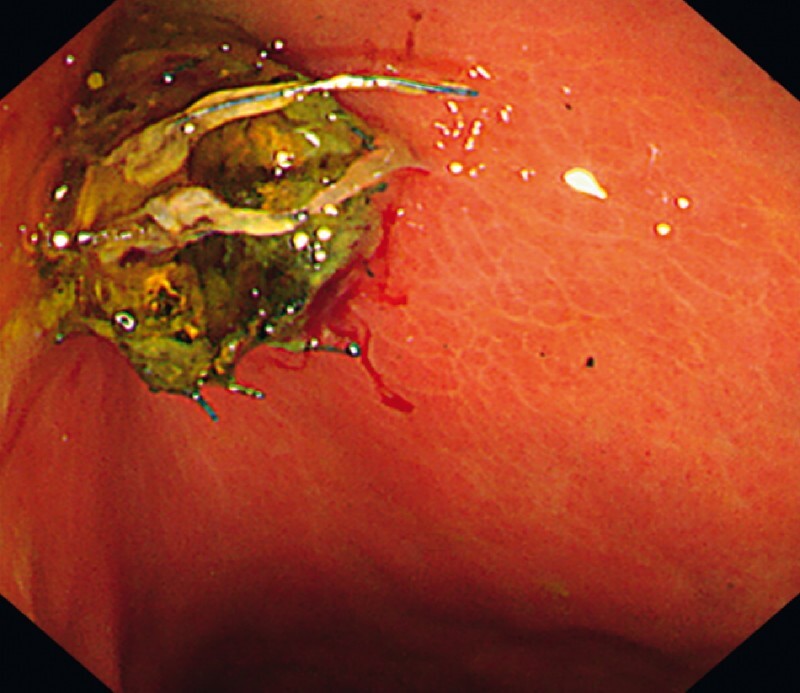
*Case 1.* Endoscopic view of the trimmed SEMS. The stent cross-section was clean because of the stable positioning for APC cutting.

**Video 1** A novel re-intervention technique for metal stent dysfunction following interventional endoscopic ultrasonography (EUS)-guided procedures: a two-channel scope is used, with a forceps stabilizing the stent position as it is trimmed using argon plasma coagulation (APC).


*Case 2.* A 48-year-old man with an advanced pancreatic cancer suffered from jaundice. Despite needle-knife precutting, biliary cannulation was challenging and EUS-guided antegrade stenting had been performed, deploying an uncovered laser-cut type SEMS across the papilla. The stent contacted the duodenal wall, leading to duodenal ulcer, stent occlusion, food impaction, and cholangitis. Therefore we trimmed the SEMS, using the two-channel scope. Laser-cut type SEMS are brittle and easily tangle when trimmed with APC, making it difficult to cut the stent as desired. Use of the two-channel scope enabled fixing the position of the SEMS, by grasping with forceps, during the APC cutting ( Fig. 4 ). Thus the stent could be cleanly trimmed while avoiding cauterization of the duodenal mucosa ( Fig. 5 , Video 1 ).

**Fig. 4:**
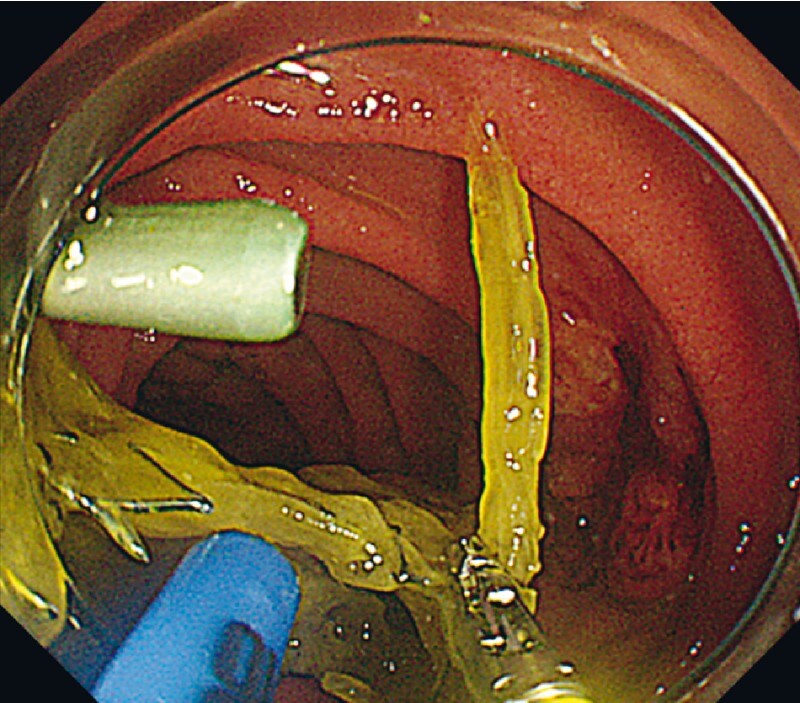
*Case 2.* Trimming of SEMS. Laser-cut type SEMS are brittle and easily tangled during trimming. Use of the forceps with the two-channel scope allowed the stent to be left in place while retaining its shape.

**Fig. 5:**
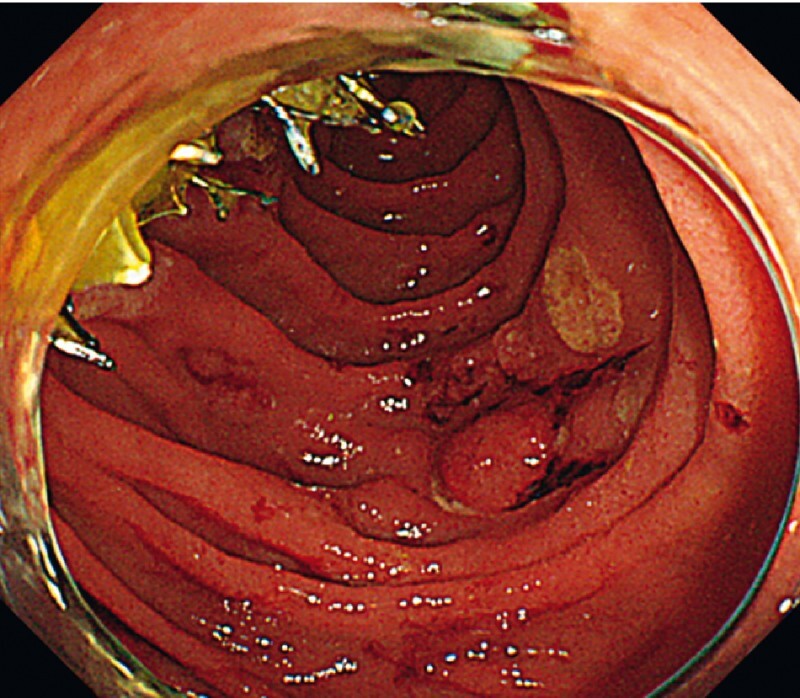
*Case 2.* The trimmed SEMS. Because the stent was cut to the appropriate length, it no longer interfered with the passage of food residues or came in contact with the duodenal mucosa.

If the target SEMS can be accessed by a two-channel scope, this may be a useful re-intervention technique for trimming SEMS.

Endoscopy_UCTN_Code_CPL_1AL_2AD

## Notes

Endoscopy E-Videos

https://eref.thieme.de/e-videos
*E-Videos* is an open access online section of the journal *Endoscopy* , reporting on interesting cases and new techniques in gastroenterological endoscopy. All papers include a high-quality video and are published with a Creative Commons CC-BY license. Endoscopy E-Videos qualify for HINARI discounts and waivers and eligibility is automatically checked during the submission process. We grant 100% waivers to articles whose corresponding authors are based in Group A countries and 50% waivers to those who are based in Group B countries as classified by Research4Life (see: https://www.research4life.org/access/eligibility/ ). This section has its own submission website at https://mc.manuscriptcentral.com/e-videos
